# A Low-Offset Sense Amplifier with Self-Adaptive Calibration and Dynamic Body-Biased Mitigation Technology for Enhanced SRAM Read Performance

**DOI:** 10.3390/mi17050591

**Published:** 2026-05-11

**Authors:** Yulan Liu, Yibo Hu, Han Xiao, Yuanzhen Liu, Jing Chen

**Affiliations:** 1 State Key Laboratory of Materials for Integrated Circuits, Shanghai Institute of Microsystem and Information Technology, Chinese Academy of Sciences, Shanghai 200050, China; ylliu@mail.sim.ac.cn (Y.L.); ybhu@mail.sim.ac.cn (Y.H.); hanx@mail.sim.ac.cn (H.X.); yzliu@mail.sim.ac.cn (Y.L.); 2 School of Electronic, Electrical and Communication Engineering, University of Chinese Academy of Sciences, Beijing 100049, China

**Keywords:** sense amplifier (SA), static random-access memory (SRAM), threshold voltage mismatch, offset calibration, body bias

## Abstract

Offset voltage ($V_{\mathrm{OS}}$) is a critical parameter of sense amplifiers (SAs), determining both the read reliability and performance of SRAM. This paper proposes SC-DISBSA, a low-$V_{\mathrm{OS}}$ SA that combines self-adaptive calibration with dynamic body bias technology. Based on the linear relationship between the transfer gate voltage and $V_{\mathrm{OS}}$, a three-step self-adaptive calibration algorithm is established. Supported by the calibration control circuit, this approach quantitatively calibrates circuit mismatch while dynamic body bias further suppresses remaining variations. Under a 28 nm CMOS process, the $V_{\mathrm{OS}}$ standard deviation ($\sigma_{\mathrm{OS}}$) of SC-DISBSA remains below 3.1 mV across a 0.7 V to 1.1 V supply range, representing reductions of 49.9% and 69.3% compared to the voltage-latch SA (VLSA) and current-latch SA (CLSA), respectively. At a typical case (TT/0.9 V/27 °C) with a BL differential ($\Delta V_{\mathrm{BL}}$) of $6\sigma_{\mathrm{OS}}$, SC-DISBSA reduces the required bitline discharge delay by 51.7% and improves average read sensing power by 24.9% compared to VLSA. By adopting an non-conventional bitline power supply strategy, SC-DISBSA decreases worst case (FF/1.1 V/125 °C) static power by 36.8% relative to VLSA. Additionally, it reduces gate area by 18.9%. Overall, SC-DISBSA effectively optimizes SRAM read latency and power efficiency.

## 1. Introduction

Static random-access memory (SRAM) is extensively utilized for Level 1–3 caches in systems-on-chips (SoCs), benefiting from its high speed, superior reliability, and high CMOS process compatibility [1,2,3,4]. Within the SRAM read path, the sense amplifier (SA) critically effects both access time and energy consumption. During read operations, the bitcell discharges through one bitline (BL) through its access transistor, while the complement bitline (BLB) remains at the supply voltage (VDD). This establishes a small BL differential (Δ$V_{\mathrm{BL}}$), transmitted to the SA through the column multiplexer. Research [5] demonstrates that the minimum $\Delta V_{\mathrm{BL}}$ exhibits positive correlation with both read latency and power consumption. For reliable data retrieval, the minimum $\Delta V_{\mathrm{BL}}$ must surpass the offset voltage ($V_{\mathrm{OS}}$) of SAs. Excessive $V_{\mathrm{OS}}$ not only extends BL discharge delay ($T_{\mathrm{BL}}$) but also increases read power significantly. In low-voltage applications, the SA further constrains the SRAM’s minimum supply [6,7,8].

**Figure 1:**
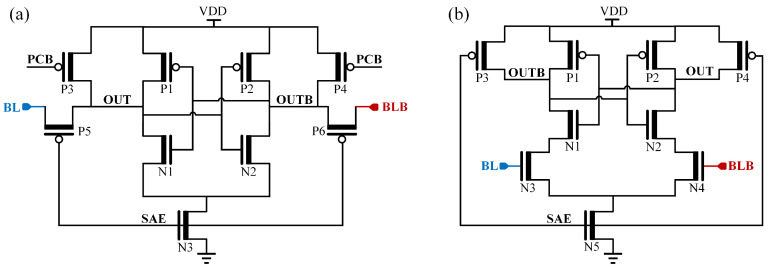
Classic latch-type SAs. (**a**) VLSA. (**b**) CLSA.

The latch-type SA is widely adopted in memory designs owing to its simple structure, high speed, reliability, and power efficiency [9,10]. Its fundamental configuration comprises cross-coupled inverter pairs that enable high-gain, rapid differential amplification via positive feedback. Figure 1 depicts two classic implementations: the voltage-latched SA (VLSA) [11] and current-latched SA (CLSA) [12]. The $V_{\mathrm{OS}}$ primarily manifests from threshold voltage mismatch ($\Delta V_{\mathrm{TH}}$) in critical sensing transistors, output capacitance variations, and layout asymmetries [10,13,14], among which $\Delta V_{\mathrm{TH}}$ constitutes the dominant contributor [15,16]. Studies by Singh [16] and Shah [14] established $V_{\mathrm{OS}}$ models for VLSA and CLSA, confirming that with VDD precharged BLs, the latch-NMOS pair serves as the pivotal sensing element where $\Delta V_{\mathrm{TH}}$ determines the $V_{\mathrm{OS}}$. Woo et al. [17] subsequently developed a comprehensive $V_{\mathrm{OS}}$ model incorporating secondary transistor effects. In advanced technology nodes, aggressive device scaling exacerbates process variations, significantly increasing random Δ$V_{\mathrm{TH}}$ and consequently worsening $V_{\mathrm{OS}}$ [18,19]. This deterioration compromises the SA’s ability to detect minute voltage differences, ultimately elevating read failure risks [10,13].

The Pelgrom mismatch model [11,20] demonstrates that the $V_{\mathrm{OS}}$ exhibits inverse proportionality to the square root of gate area. Although enlarging device dimensions offers the most direct approach for Δ$V_{\mathrm{TH}}$ reduction, it provides limited improvement while introducing significant parasitic effects that incur additional delay and power penalties. Previous research has extensively investigated various $V_{\mathrm{OS}}$ cancellation and compensation techniques [20,21,22,23,24,25,26,27,28,29,30,31,32]. The first common category employs additional capacitors or transistors to store the offset polarity and realize multi-stage quantitative calibration [23,24,25,26,27]. Ref. [27] integrated a switched-capacitor voltage booster to enhance both differential and common mode BL voltages in VLSA, showing a decrease of 23% $V_{\mathrm{OS}}$ at VDD = 0.3 V, albeit with 12% area overhead and complex timing requirements. Another method utilizes body effect or internal feedback mechanisms for adaptive mismatch compensation [28,29,30,31,32]. The body-biased calibrated SA [32] reduces $V_{\mathrm{OS}}$ by 49% relative to CLSA, with only a 3.5% increase in the overall SRAM area. Multi-phase timing control used to adjust transistor connections and optimize circuit matching is another common strategy. Ref. [33] employs multi-phase capacitors to cause a 50% $V_{\mathrm{OS}}$ reduction at 0.5V VDD compared to VLSA, requiring 3.2% additional area. Furthermore, ref. [21] optimized CLSA offset using precise precharge technology, achieving a 7.6% improvement in equivalent gate area.

This work targets Δ$V_{\mathrm{TH}}$ in critical sensing transistors, proposing a SC-DISBSA structure that employs a three-step self-adaptive calibration technique through gate-biased control for a differential-input supplied body-biased SA (DISBSA). Designed for high-speed SRAM employing symmetric bitcells such as 6T and 10T, the SC-DISBSA features three fundamental characteristics:

- Since the BL pair serves both as differential inputs and power supply for the differential branches, the dynamic power of SA is negligible.
- By exploiting the dynamic body effect to mitigate circuit mismatch, the proposed designs enhance the sensing capability.
- By storing the offset polarity and quantitatively modulating the gate voltage of the transmission transistors, the $V_{\mathrm{OS}}$ distribution is compressed toward the mean ($\mu_{\mathrm{OS}}$), effectively reducing the $V_{\mathrm{OS}}$.

Simulation results verify that the proposed SC-DISBSA maintains stable and superior performance across PVT variations, significantly enhancing SRAM read performance. The remainder of this paper is organized as follows: Section 2 details the circuit structure, operating principles, and calibration methodology. Section 3 analyzes performance under various conditions and compares the proposed SA against conventional and state-of-the-art designs. Section 4 summarizes the work.

## 2. Proposed Circuit

### 2.1. Proposed SC-DISBSA and SC-DISSA

**Figure 2:**
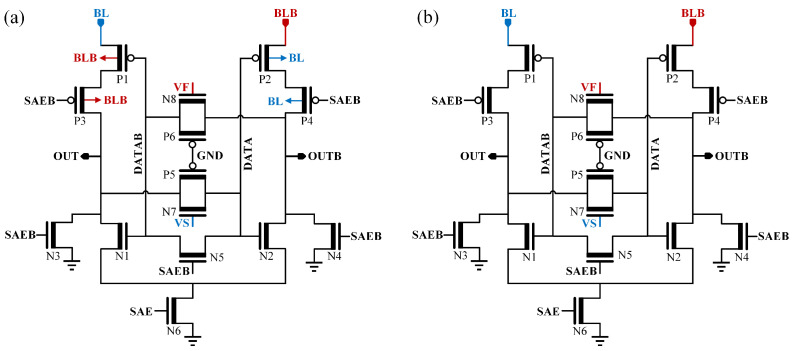
Schematic of (**a**) SC-DISBSA and (**b**) SC-DISSA.

Figure 2a illustrates the proposed SC-DISBSA. Unlike conventional SAs, BL and BLB act not only as differential inputs but also as power sources for the differential branches. Transistors N3–N5 provide pre-discharge paths for OUT and OUTB nodes. During the disable phase (signal SAE is low), P3 and P4 isolate BL/BLB from OUT/OUTB to prevent voltage interference. The circuit features two pseudo-inverters formed by the N1/P1/P3 and N2/P2/P4 clusters. The body terminals of P1/P3 and P2/P4 are tied to BLB and BL, respectively. These pseudo-inverters form a latch configuration via head-to-tail connections through transmission gates (TG: N7/P5 and N8/P6), while the gates of P5 and P6 are grounded, N7/N8 gates receive external calibration voltages VS/VF. This calibration methodology is detailed in the following subsection. Due to the pre-discharge strategy, P1–P4 act as the critical sensing devices for pulling up the output nodes. Consequently, the $V_{\mathrm{TH}}$ mismatch among these transistors primarily determines the $V_{\mathrm{OS}}$.

(1) $$\begin{array}{c} V_{\mathrm{TH}}=V_{T0}+\gamma \left(\sqrt{|\left(-2\varphi_{F}\right)+V_{\mathrm{SB}}|-\sqrt{-2\varphi_{F}}}\right) \end{array}$$

Body bias alters the device current by affecting the MOSFET’s $V_{\mathrm{TH}}$. The $V_{\mathrm{TH}}$ model is given by Equation (1), where $V_{T0}$ is the intrinsic threshold voltage in the absence of body bias, γ is the body effect coefficient, and $V_{\mathrm{SB}}$ is the bias voltage from source to body. Forward body bias ($V_{\mathrm{SB}}$ > 0) reduces the absolute value of PMOS $V_{\mathrm{TH}}$ (|$V_{\mathrm{THP}}$|), while reverse body bias ($V_{\mathrm{SB}}$ < 0) increases |$V_{\mathrm{THP}}$|.

In 28 nm CMOS process with VDD = 0.9 V, a 9 mV |$V_{\mathrm{THP}}$| reduction in saturated PMOSs when $V_{\mathrm{SB}}$ varies from −40 mV to 40 mV. As body effect diminishes with technology scaling and becomes negligible in advanced 3D FETs (FinFETs, GAA FETs), the differential-input-supplied SA (DISSA) with self-adaptive calibration (SC-DISSA) in Figure 2b offers broader applicability.

**Figure 3:**
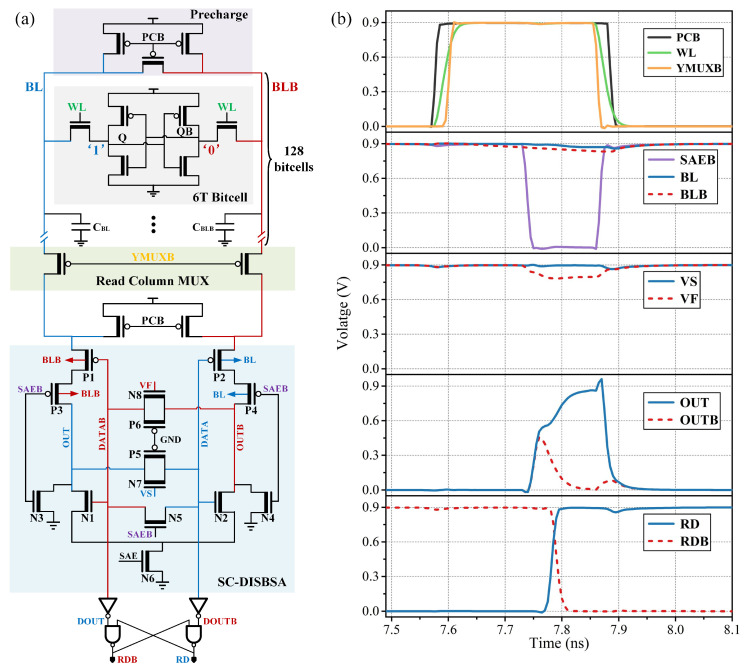
(**a**) 6T SRAM read path using the SC-DISBSA scheme. (**b**) Operational waveforms of the SC-DISBSA.

Figure 3a illustrates the SRAM read path using SC-DISBSA. In addition to the SA and memory column, the configuration includes the BL/BLB precharge circuit, read column multiplexer, and SR latch. Each BL/BLB is connected to 128 6T bitcells. The BL capacitive loads ($C_{\mathrm{BL}}$ and $C_{\mathrm{BLB}}$) incorporate the effects of the write column multiplexer and write driver, which are omitted from the figure for clarity. Before the read operation, OUT/OUTB nodes are pre-discharged to ground, while BL/BLB nodes and VF/VS are pulled up to VDD. The activation of WL and the read column select signal (YMUXB) marks the onset of the read operation. The BLB node of the 6T bitcell storing a “0” discharges slowly, whereas the BL node remains at a high level. This differential voltage, $|\Delta V_{\mathrm{BL}}=V_{\mathrm{BL}}-V_{\mathrm{BLB}}|$, subjects transistors P1 and P2 to forward and reverse body biases, respectively. Once Δ$V_{\mathrm{BL}}$ reaches a sufficient margin, the SAE signal activates. During the sensing phase, VS and VF are connected to calibration voltages latched from the self-adaptive calibration operation (in Figure 3b, VS = VDD and VF = Vref1) to mitigate inherent mismatches. These details will be discussed in the next subsection. At this stage, P1 and P3 exhibit similar forward body biases, decreasing |$V_{\mathrm{THP}1}$| ($V_{\mathrm{THP}1}$ denotes the equivalent $V_{\mathrm{THP}}$ of the series structure formed by P1 and P3). Conversely, P2 and P4 exhibit similar reverse body biases, increasing |$V_{\mathrm{THP}2}$| ($V_{\mathrm{THP}2}$ denotes the equivalent $V_{\mathrm{TH}}$ of the series structure of P3 and P4).

The body effects of transistors P1-P4 create a current competition mechanism that favors sensing accuracy. Specifically, a decrease in |$V_{\mathrm{THP}1}$| and an increase in |$V_{\mathrm{THP}2}$| reduce the equivalent resistance of the BL branch while increasing that of the BLB branch. This accelerates the charging of the OUT node and slows that of the OUTB node, facilitating a rapid transition toward the correct result. Once the OUT node voltage exceeds the threshold of N1, it pulls OUTB down. Subsequently, the regenerative feedback of the latch unit takes effect, rapidly pulling OUT to VDD. Facilitated by the SR latch, the read data signals RD/RDB complete their flip. The operational waveforms are shown in Figure 3b. SC-DISSA follows a similar operating process and timing but lacks the body-effect modulation of P1–P4.

Suppose there is a circuit mismatch in the SC-DISBSA, where Δ$V_{\mathrm{TH}}$ = $|V_{\mathrm{THP}1}\left|\;-\;\right|V_{\mathrm{THP}2}|$ < 0, causing OUT to charge faster than OUTB. When the differential input is $V_{\mathrm{BL}}$ < $V_{\mathrm{BLB}}$ (Δ$V_{\mathrm{BL}}$ = $V_{\mathrm{BL}}-V_{\mathrm{BLB}}$ < 0), the mismatch Δ$V_{\mathrm{TH}}$ < 0 degrades the sensing ability, potentially leading to an incorrect scenario where OUT erroneously transitions to a high level before OUTB. Conversely, when Δ$V_{\mathrm{BL}}$ > 0, a mismatch of Δ$V_{\mathrm{TH}}$ < 0 facilitates the correct transition of output nodes. The same principle applies to cases when Δ$V_{\mathrm{TH}}$ > 0. Therefore, the interplay between Δ$V_{\mathrm{TH}}$ and Δ$V_{\mathrm{BL}}$ polarities is summarized as follows: (1) If their polarities are consistent, the mismatch impairs the sensing ability and is defined as an unfavorable mismatch. (2) If their polarities are opposite, the mismatch accelerates the correct transition and is defined as a favorable mismatch. SC-DISBSA utilizes dynamic body bias to suppress unfavorable mismatches and enhance favorable ones, effectively mitigating the impact of device variations and enhancing the sensing ability.

### 2.2. Gate-Biased Self-Adaptive Calibration

**Figure 4:**
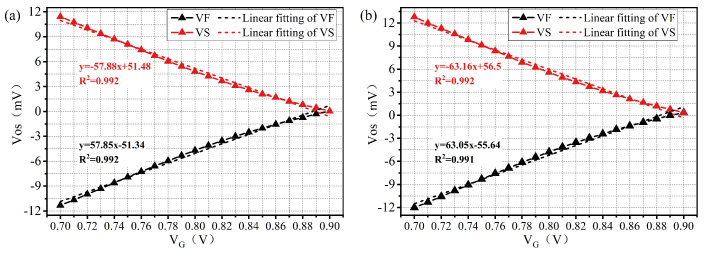
$V_{\mathrm{OS}}$ versus VF/VS transfer curves: (**a**) SC-DISBSA and (**b**) SC-DISSA.

Modulating device current via gate voltage effectively mitigates circuit mismatches. As shown in Figure 4, the $V_{\mathrm{OS}}$-VG transfer curves demonstrate that VF and VS exert a symmetrical linear influence on the $V_{\mathrm{OS}}$ of both SC-DISBSA and SC-DISSA. Specifically, $V_{\mathrm{OS}}$ increases linearly with VF and decreases linearly with VS. These transfer curves in Figure 4 fit linear functions with coefficients of determination ($R^{2}$) exceeding 0.99. To shift $V_{\mathrm{OS}}$ of SC-DISBSA by a magnitude of M, the required gate voltage can be determined by setting y = M. Combined with digital control, this approach enables quantitative $V_{\mathrm{OS}}$ calibration.

**Figure 5:**
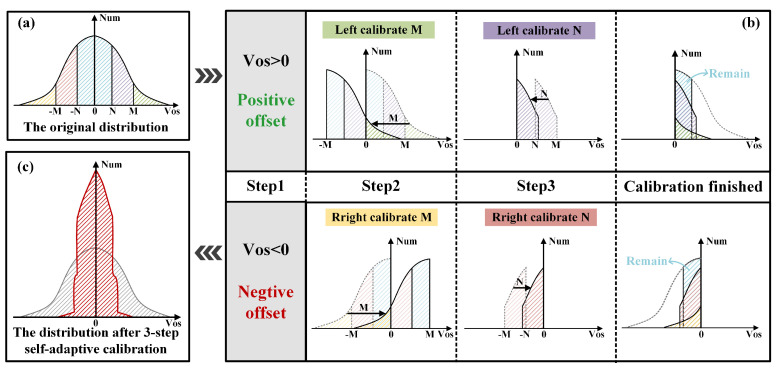
(**a**) The distribution of $V_{\mathrm{OS}-\mathrm{ori}}$. (**b**) Three-step self-adaptive calibration procedure. (**c**) The distribution of $V_{\mathrm{OS}-\mathrm{cali}}$.

Based on the preceding analysis, this work proposes a three-step self-adaptive calibration technique. By leveraging the distribution characteristics of $V_{\mathrm{OS}}$, this technique reshapes the distribution through region partitioning and quantitative translation toward $\mu_{\mathrm{OS}}$. Figure 5a shows the original $V_{\mathrm{OS}}$ ($V_{\mathrm{OS}-\mathrm{ori}}$) distribution, which follows the Gaussian model described in Equation (2).

(2) $$\begin{array}{c} V_{\mathrm{OS}-\mathrm{ori}}=\frac{1}{\sqrt{2\pi }\sigma_{\mathrm{OS}}}e^{-\frac{{\left(x-\mu_{\mathrm{OS}}\right)}^{2}}{2\sigma_{\mathrm{OS}}^{2}}} \end{array}$$

Figure 5b illustrates the self-adaptive calibration process, which requires three cycles to complete: offset polarity detection and storage, a coarse shift of magnitude M, and a fine shift of magnitude N. This multi-step approach determines the optimal shift and the required gate voltage to compensate for the current mismatch as detailed below:

- Step 1: Detect and store the $V_{\mathrm{OS}}$ polarity. Positive polarity triggers a left shift, while negative polarity initiates a right shift.
- Step 2: Execute a shift of magnitude M toward $\mu_{\mathrm{OS}}$. For $V_{\mathrm{OS}-\mathrm{ori}}$ within (−∞,−M)∪(M,+∞), if the polarity remains unchanged after the shift, the coarse calibration is considered successful. If $V_{\mathrm{OS}-\mathrm{ori}}$ falls within [−M,M], the shift would cause a polarity reversal, indicating overcompensation. In this case, the shift should be canceled.
- Step 3: Execute a shift of magnitude N toward $\mu_{\mathrm{OS}}$. For $V_{\mathrm{OS}-\mathrm{ori}}$ within [−M,−N)∪(N,+M], if the polarity remains unchanged, the fine shift is appropriate and the process terminates. For $V_{\mathrm{OS}-\mathrm{ori}}$ within [−N,N], even a small shift of N results in polarity reversal. Therefore, no calibration should be performed for circuit mismatches within this interval.

Figure 5c illustrates the distribution of the calibrated $V_{\mathrm{OS}}$ ($V_{\mathrm{OS}-\mathrm{cali}}$), which is significantly narrower and more concentrated, as expressed by Equation (3).

(3) $$\begin{array}{cc} \; & V_{\mathrm{OS}-\mathrm{cali}}=\left\{\begin{array}{cc} \frac{1}{\sqrt{2\pi }\sigma_{\mathrm{OS}}}\left(e^{-\alpha }+e^{-\beta }+e^{-\gamma }\right), & |x|\leq N\\ \frac{1}{\sqrt{2\pi }\sigma_{\mathrm{OS}}}\left(e^{-\alpha }+e^{-\beta }\right), & N<|x|\leq M-N\\ \frac{1}{\sqrt{2\pi }\sigma_{\mathrm{OS}}}\left(e^{-\alpha }+e^{-\beta }\right), & |x|>M-N \end{array}\right.\\ \; & \alpha =\frac{{\left(|x|+M\right)}^{2}}{2\sigma_{\mathrm{OS}}^{2}},\;\beta =\frac{{\left(|x|+N\right)}^{2}}{2\sigma_{\mathrm{OS}}^{2}},\;\gamma =\frac{x^{2}}{2\sigma_{\mathrm{OS}}^{2}} \end{array}$$

**Figure 6:**
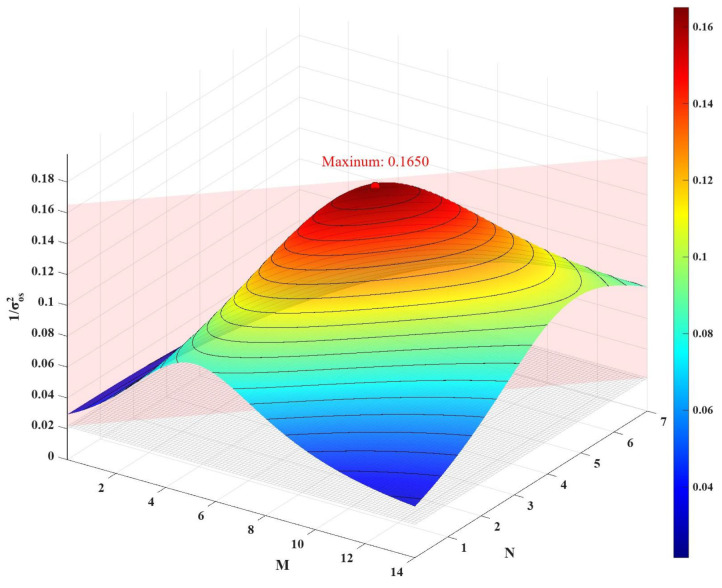
Three-dimensional relationship of M, N, and $1/\sigma_{\mathrm{OS}}^{2}$ for SC-DISBSA when initial $\sigma_{\mathrm{OS}}$ = 5.88 mV.

At VDD = 0.9 V, the uncalibrated SC-DISBSA exhibits a $\sigma_{\mathrm{OS}}$ of 5.88 mV. Based on Equation (3), MATLAB R2026a is employed to analyze the relationship between M, N, and 1/$\sigma_{\mathrm{OS}}^{2}$ given $\sigma_{\mathrm{OS}}$ = 5.88 mV. As shown in Figure 6, with M = 7.8 and N = 3.47, the theoretically optimal $\sigma_{\mathrm{OS}}$ is reduced to 2.46 mV. According to the fitting function in Figure 4, the gate voltages required to achieve shifts of M and N are 750 mV and 825 mV, respectively. Similarly, the initial $\sigma_{\mathrm{OS}}$ of SC-DISSA is 6.53 mV, which improves to 2.73 mV under optimal configurations (M = 8.65, N = 3.9), corresponding to gate voltages of 755 mV and 830 mV.

### 2.3. Calibration Control Circuit

**Figure 7:**
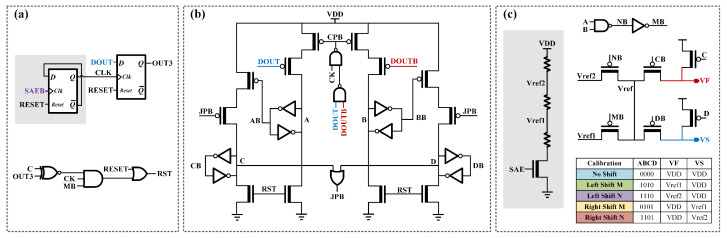
Three-step self-adaptive calibration control circuit. (**a**) Reset control circuit. (**b**) $V_{\mathrm{OS}}$ polarity latch and calibration magnitude selection circuit. (**c**) Calibration voltage generation and selection circuit.

The implementation of the self-adaptive calibration requires the control circuit shown in Figure 7. Figure 7a depicts the reset control module for initialization or no-shift operations. Figure 7b shows the $V_{\mathrm{OS}}$ polarity latch and magnitude determination circuit. Signals C and D store the polarity to dictate the calibration direction, while signals A and B control the gate voltages (Vref1, Vref2, and VDD) to set the calibration magnitude. The reference voltage generation and selection circuit are shown in Figure 7c. To minimize area overhead, the gray region in Figure 7 includes the reference voltage generator and Step 3 of the detection circuit, which can be shared across multiple SC-DISBSA and SC-DISSA units. Conversely, the remaining components, which store specific offset polarities and voltage configurations, are dedicated to each SA and cannot be shared. The possible states of the control circuit are summarized in the table in Figure 7c.

**Figure 8:**
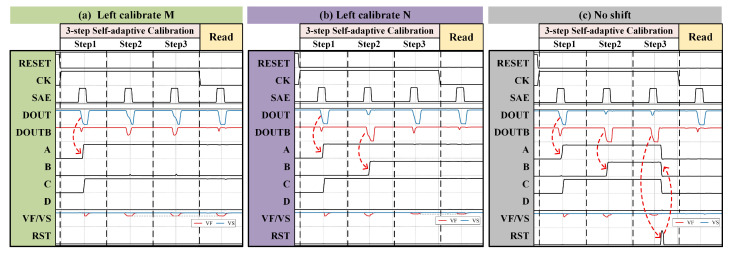
The following operational waveforms of the calibration control circuit when Δ$V_{\mathrm{TH}}$ > 0 may occur: (**a**) Left shift M. (**b**) Left shift N. (**c**) No shift.

Assuming $\Delta V_{\mathrm{TH}}$ > 0 in the SC-DISBSA, OUTB may reach the switching threshold of pseudo-inverters before OUT does. In this positive $V_{\mathrm{OS}}$ scenario, VS should be set to VDD and VF to Vref to increase the equivalent $V_{\mathrm{TH}}$ of the P2 and P4 series structure. Depending on the mismatch degree, three calibration outcomes illustrated in Figure 8 may occur: left shift M, left shift N, or no shift. Prior to calibration, signals A–D are reset to “0”, while signals VS and VF are reset to VDD, representing a zero-shift state. Throughout calibration cycles, BL and BLB are continuously precharged to VDD.

- Step 1: With A–D initialized to “0”, VF = VS = VDD. Upon SAE activation, the $V_{\mathrm{OS}}$ polarity is determined by DOUT and DOUTB. If DOUT/DOUTB = “0”/“1”, a positive $V_{\mathrm{OS}}$ is identified, triggering a sequential pull-up of A and C to “1”.
- Step 2: With A = C = “1” and B = D = “0”, setting VF = Vref1 and VS = VDD enables an M-magnitude left shift upon SAE activation. In Figure 8a, unchanged DOUT/DOUTB confirms that the M-compensation is sufficient, and signals A–D are latched to finalize calibration. Conversely, in Figure 8b,c, DOUT/DOUTB flips to “1”/“0”, indicating M overcompensation, which pulls B to “1”.
- Step 3: With A = B = C = “1” and D = “0”, setting VF = Vref2 and VS = VDD enables an N-magnitude shift upon SAE activation. If DOUT/DOUTB matches Step 1 like in Figure 8b, the compensation is validated. In contrast, Figure 8c shows DOUT/DOUTB inversion relative to Step 1, indicating N overcompensation. The reset control circuit asserts the RST signal to clear A–D to ‘0’ and reset VF/VS to VDD.

In subsequent read cycles, the reference voltages stored in the calibration control circuit are applied to VF and VS nodes at the onset of the sensing phase.

## 3. Simulation Results and Analysis

### 3.1. $V_{\mathrm{OS}}$

**Figure 9:**
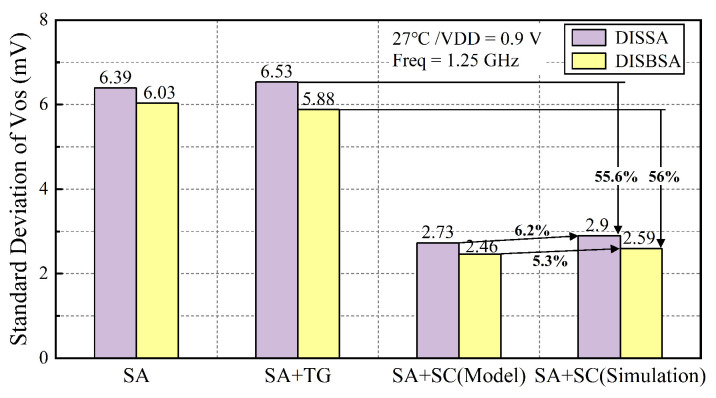
$\sigma_{\mathrm{OS}}$ comparison among SC-DISBSA, SC-DISSA, and their topologies at VDD = 0.9 V.

The proposed SA structures were integrated into an SRAM macro with an array of 128 rows and 64 columns, utilizing an 8-to-1 read column multiplexer. To evaluate the $V_{\mathrm{OS}}$ of SC-DISBSA and its variants, 1000-point Monte Carlo (MC) simulations were performed at VDD = 0.9 V and Freq = 1.25 GHz. The simulation accounted for both global process variations and random device mismatch using a random sampling method. As illustrated in Figure 9, “SA” denotes the DISBSA/DISSA where transmission gates are replaced by metal interconnects, while “SA+TG” represents the structures with VF and VS nodes tied to VDD. Simulation results show that the $\sigma_{\mathrm{OS}}$ reduction for DISBSA+TG compared to DISBSA is negligible, while the $\sigma_{\mathrm{OS}}$ of SC-DISSA is effectively reduced by 55.6% compared to DISSA+TG, and the $\sigma_{\mathrm{OS}}$ of SC-DISBSA is reduced by 56% compared to DISBSA+TG. These results demonstrate that the three-step self-adaptive calibration significantly suppresses the offset. Furthermore, the deviation between the simulated $\sigma_{\mathrm{OS}}$ and the theoretically optimal calibration value is less than 6.2%, verifying the calibration model accuracy. Additionally, the $\sigma_{\mathrm{OS}}$ of DISBSA+TG improves by 10% over DISSA+TG, and SC-DISBSA improves by 10.7% over SC-DISSA. These data validate that the dynamic body bias helps to mitigate mismatch, consistent with the previous analysis. To conclude, the self-adaptive calibration serves as the principal mechanism for mismatch reduction, with dynamic body bias acting as a supplementary contributor to offset mitigation.

**Figure 10:**
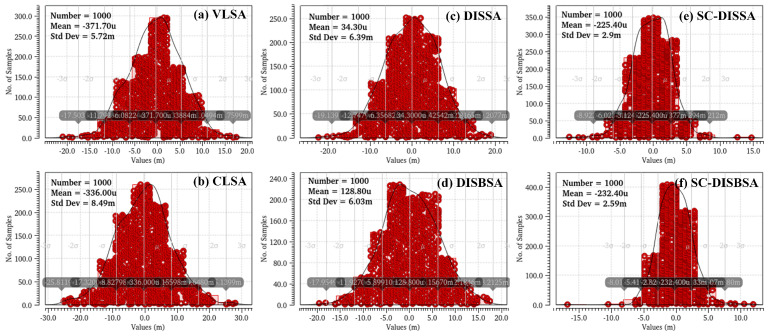
Distribution of $V_{\mathrm{OS}}$ at VDD = 0.9 V and Freq = 1.25 GHz. (**a**) VLSA. (**b**) CLSA. (**c**) DISSA. (**d**) DISBSA. (**e**) SC-DISSA. (**f**) SC-DISBSA.

Under identical conditions, we compare the $\sigma_{\mathrm{OS}}$ of proposed designs against VLSA and CLSA in Figure 10. Relative to CLSA, DISSA and DISBSA reduce $\sigma_{\mathrm{OS}}$ by 23.8% and 28.1%, respectively, while SC-DISSA and SC-DISBSA achieve greater reductions of 65.8% and 69.5%, respectively. Contrasted with VLSA, DISSA and DISBSA show $\sigma_{\mathrm{OS}}$ increases of 11.7% and 5.4%, whereas SC-DISSA and SC-DISBSA demonstrate significant reductions of 49.3% and 54.7%. Furthermore, SC-DISSA and SC-DISBSA exhibit $V_{\mathrm{OS}}$ distributions with higher peaks and narrower spreads, consistent with prior analysis.

**Figure 11:**
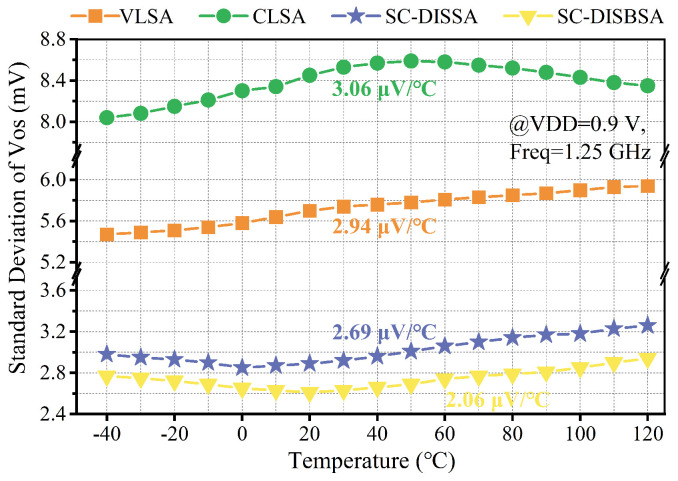
Trends of $\sigma_{\mathrm{OS}}$ versus temperature (from −40 °C to 120 °C) at VDD = 0.9 V.

Figure 11 illustrates the $\sigma_{OS}$ temperature dependence from −40 °C to 120 °C at VDD = 0.9 V. While CLSA, SC-DISSA, and SC-DISBSA exhibit non-monotonic $\sigma_{OS}$ variations, VLSA shows a monotonic increase with temperature. Overall, the proposed SAs maintain lower temperature drift coefficients than conventional latch-type designs.

**Figure 12:**
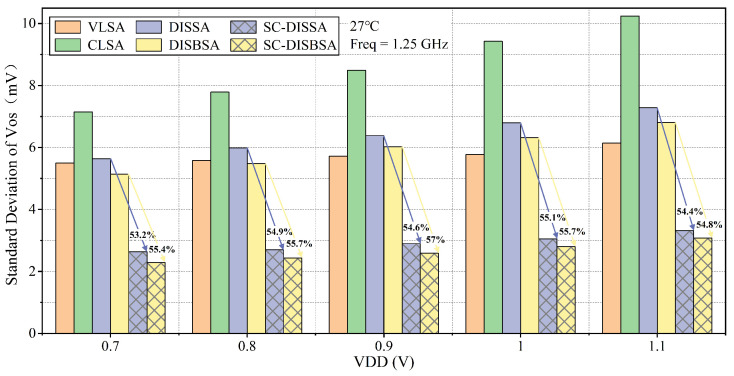
Comparison of $\sigma_{\mathrm{OS}}$ across various supply voltages at 27 °C and Freq = 1.25 GHz.

The supply stability of $V_{\mathrm{OS}}$ is validated through 1000-point MC simulations across a range of 0.7 V–1.1 V. In Figure 12, SC-DISBSA attains $\sigma_{\mathrm{OS}}$ reductions, exceeding DISBSA and VLSA by 54.8% and 49.9%, respectively. Similarly, SC-DISSA demonstrates $\sigma_{\mathrm{OS}}$ improvements, surpassing DISSA by 53.2% and VLSA by 46%. Throughout this supply range, the $\sigma_{\mathrm{OS}}$ of SC-DISBSA and SC-DISSA remains below 3.1 mV and 3.3 mV, with SC-DISBSA achieving an average 9.9% reduction compared to SC-DISSA.

In practical operation, noise interferes with the sensing process and may lead to erroneous results. This work accounts for the simultaneous effects of supply voltage, process corners, and temperature on the input-referred noise voltage ($V_{n,\mathrm{in}}$). As differential comparators, all four SA structures effectively suppress external common-mode noise. Simulation results indicate that within the 0.7–1.1 V range, the standard deviations of $V_{n,\mathrm{in}}$ ($\sigma_{n,\mathrm{in}}$) for SC-DISBSA and SC-DISSA are 1 mV and 1.04 mV, respectively. While these reflect increase over VLSA, they represent a notable improvement over CLSA. Nevertheless, the ($\sigma_{n,\mathrm{in}}$) for all SAs remain below 1.5 mV. To ensure a high sensing yield, a minimum input voltage differential ($\Delta V_{\mathrm{in}}$) must be established based on $V_{\mathrm{OS}}$ and $V_{n,\mathrm{in}}$. A theoretical sensing yield exceeding 99.99% is achievable when $\Delta V_{\mathrm{in}}$ > $4.5\sqrt{\sigma_{\mathrm{OS}}^{2}+\sigma_{n,\mathrm{in}}^{2}}$. In this design, $\Delta V_{\mathrm{in},\min}$ is defined as $6\sigma_{\mathrm{OS}}$ (significantly greater than $4.5\sqrt{\sigma_{\mathrm{OS}}^{2}+\sigma_{n,\mathrm{in}}^{2}}$) to maintain an adequate margin, which is adopted as the input condition for all subsequent simulations.

**Figure 13:**
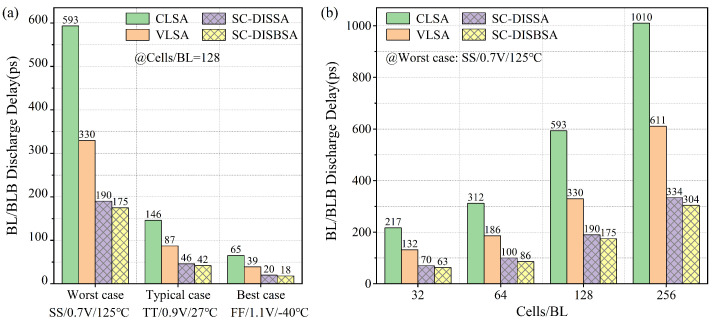
$T_{\mathrm{BL}}$ required to reach $\Delta V_{\mathrm{BL}}$ = 6$\sigma_{\mathrm{OS}}$ under (**a**) three cases and (**b**) different bitcell loads.

Figure 13a illustrates the $T_{\mathrm{BL}}$ required to reach $\Delta V_{\mathrm{BL}}$ = 6$\sigma_{\mathrm{OS}}$ under worst-case (SS/0.7 V/125 °C), typical-case (TT/0.9 V/27 °C), and best-case (FF/1.1 V/−40 °C) scenarios. Due to the linear relationship between $T_{\mathrm{BL}}$ and $\Delta V_{\mathrm{BL}}$, the $T_{\mathrm{BL}}$ required for SC-DISSA and SC-DISBSA is significantly shorter than that of CLSA. Compared to VLSA, the $T_{\mathrm{BL}}$ required for SC-DISSA and SC-DISBSA is improved by an average of 46.1% and 50.8%, respectively. Under the worst case, SC-DISBSA requires only 175 ps, whereas the VLSA and CLSA require 330 ps and 593 ps. Figure 13b depicts the $T_{\mathrm{BL}}$ trend under the worst case across BL loads ranging from 32 to 256 bitcells. The $T_{\mathrm{BL}}$ improvements for SC-DISSA and SC-DISBSA remain stable between 42% and 54% relative to VLSA. As the BL load increases from 32 to 256 bitcells, the $T_{\mathrm{BL}}$ gap between SC-DISSA/SC-DISBSA and VLSA expands from 62 ps/69 ps to 277 ps/307 ps. This indicates that low-offset sensing schemes offer more significant speed improvements at higher bitcell loads.

### 3.2. Sensing Delay and Power Consumption

**Figure 14:**
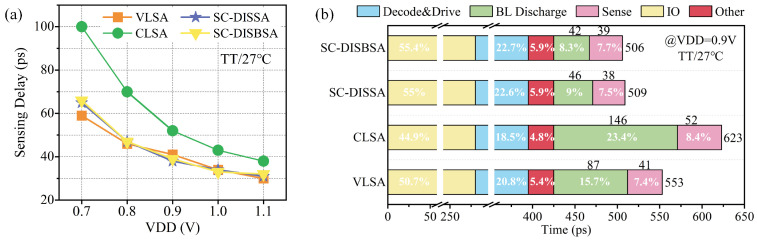
With $\Delta V_{\mathrm{BL}}$ = 6$\sigma_{\mathrm{OS}}$. (**a**) Sensing delay across different supply voltages. (**b**) Read delay distribution under the typical case.

Using $\Delta V_{\mathrm{BL}}=6\sigma_{\mathrm{OS}}$ as the input condition, Figure 14a compares the SA sensing delay ($T_{\mathrm{SA}}$) at the TT corner and 27 °C across various supply voltages. $T_{\mathrm{SA}}$ is defined as the interval from the 50% rise of the SAE signal to the 50% transition of DOUT/DOUTB signals. It is evident that the $T_{\mathrm{SA}}$ for VLSA, SC-DISBSA, and SC-DISSA remains highly consistent across the VDD range, with only a minor discrepancy at VDD = 0.7 V. This suggests that VLSA maintains a higher sensing speed at low voltages and the body effect in SC-DISBSA does not increase $T_{\mathrm{SA}}$. Figure 14b illustrates the SRAM read delay decomposition under the typical case, accounting for delays from decoding and driving as well as IO buffering. The delay breakdown shows that total read delay is primarily dominated by IO buffering, decoding and driving, and BL discharging under high loads. The contribution of $T_{\mathrm{SA}}$ remains relatively minor. Overall, the SRAM read delays for SC-DISSA and SC-DISBSA schemes are nearly identical, achieving reductions of approximately 8.2% and 18.5% compared to VLSA and CLSA schemes, respectively.

**Figure 15:**
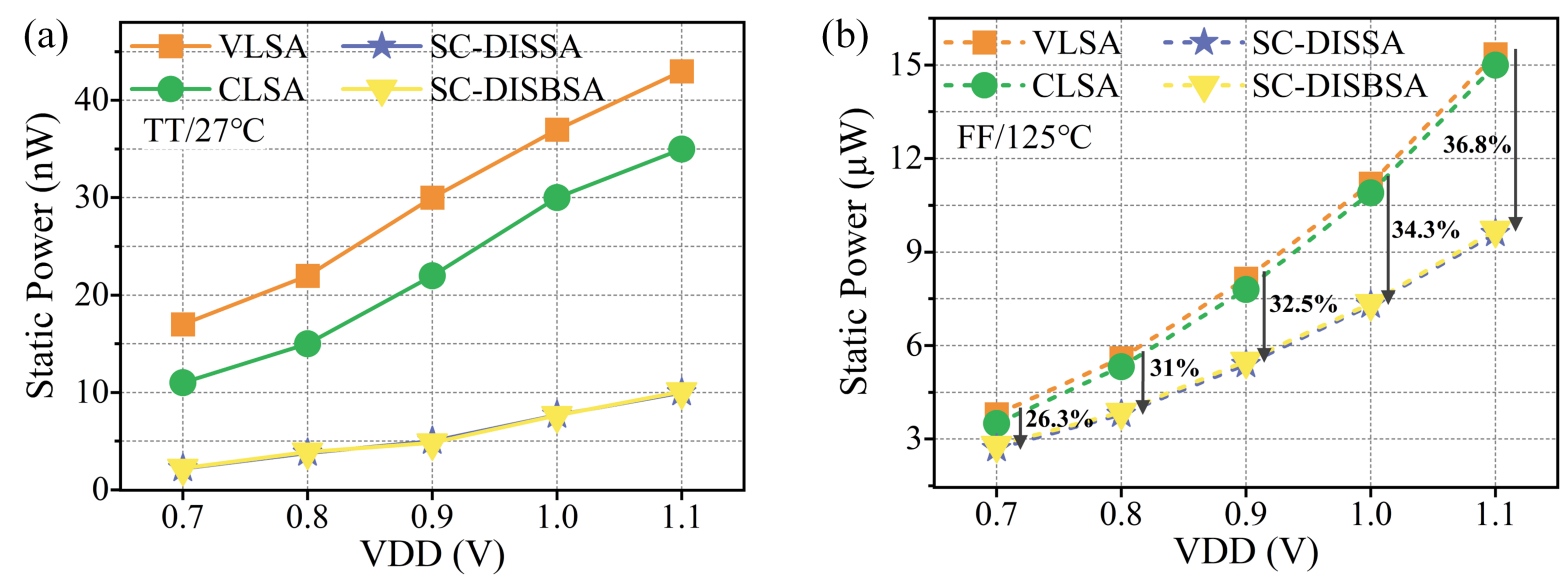
$P_{\mathrm{ST}}$ of SAs versus VDD at (**a**) TT corner and 27 °C and (**b**) FF corner and 125 °C.

A major advantage of the proposed SAs is the utilization of the BL as power supply, which reduces the power consumption. Figure 15a,b present the static power ($P_{\mathrm{ST}}$) of SAs under different conditions. VLSA consistently exhibits the highest $P_{\mathrm{ST}}$, followed by CLSA. Due to the BL power supply strategy, both SC-DISBSA and SC-DISSA achieve significant $P_{\mathrm{ST}}$ reductions. Even under the worst case, SC-DISBSA and SC-DISSA maintain a $P_{\mathrm{ST}}$ reduction of 26.3% to 36.8% compared to VLSA.

**Figure 16:**
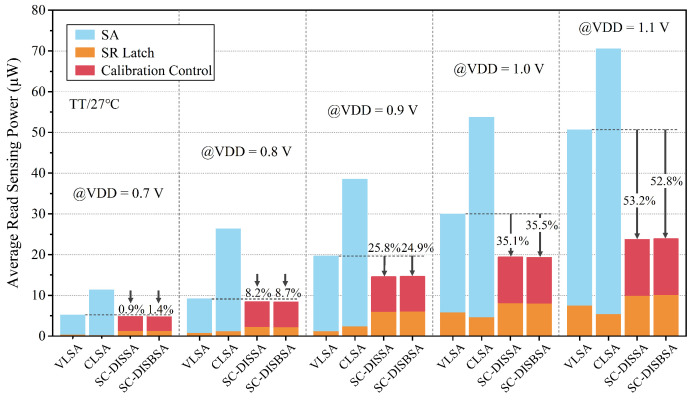
At TT corner, 27 °C, and $\Delta V_{\mathrm{BL}}$ = 6$\sigma_{\mathrm{OS}}$, the average read sensing power distribution over VDD = 0.7–1.1 V.

Figure 16 illustrates the average read sensing power ($P_{\mathrm{RS}-\mathrm{ave}}$) of the four SAs at TT corner and 27 °C. $P_{\mathrm{RS}-\mathrm{ave}}$ includes the energy consumption of the SA, SR latch, and calibration control circuit. The average dynamic power consumption of the proposed SAs is negligible for BL power supply strategy. Notably, the type of SR latch must be compatible with the initial state of output nodes. Since VLSA and CLSA employ a precharge strategy while SC-DISBSA and SC-DISSA use a pre-discharge strategy, the former pair uses NOR-type SR latches, whereas the latter requires NAND-type SR latches. Consequently, the energy consumption of SR latches in the proposed schemes is slightly higher. Nevertheless, CLSA and VLSA consume the highest $P_{\mathrm{RS}-\mathrm{ave}}$ overall. Due to structural similarities, SC-DISBSA and SC-DISSA exhibit significant and comparable energy efficiency. Moreover, the improvement increases with VDD. Compared to VLSA, the energy advantages of SC-DISBSA and SC-DISSA are marginal at 0.7 V and 0.8 V, and the improvements reach 25.8% and 24.9% at VDD = 0.9 V, respectively. This contributes significantly to reducing the overall SRAM read energy consumption.

**Figure 17:**
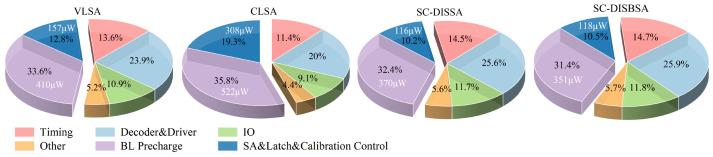
Power consumption breakdown of SRAM read operation under the typical case.

Furthermore, Figure 17 evaluates the read energy consumption of SRAM macros across the four SA schemes. BL precharging, address decoding, and driving are the primary contributors, followed by input and output buffers, with regard to timing control. BL precharging and sensing operations account for 40% to 55% of the total read power. Under the typical case, the BL precharge energy for SC-DISSA and SC-DISBSA schemes is reduced by 9.8% and 14.4% compared to VLSA, indicating that low-offset sensing schemes effectively decrease precharge energy through minimizing the BL swing. The total SRAM read power values for SC-DISSA and SC-DISBSA are 1.14 mW and 1.12 mW, representing reductions of 6.6% and 8.0% over VLSA. Overall, the proposed SAs offer a notable improvement in read power.

### 3.3. Area Overhead

**Figure 18:**
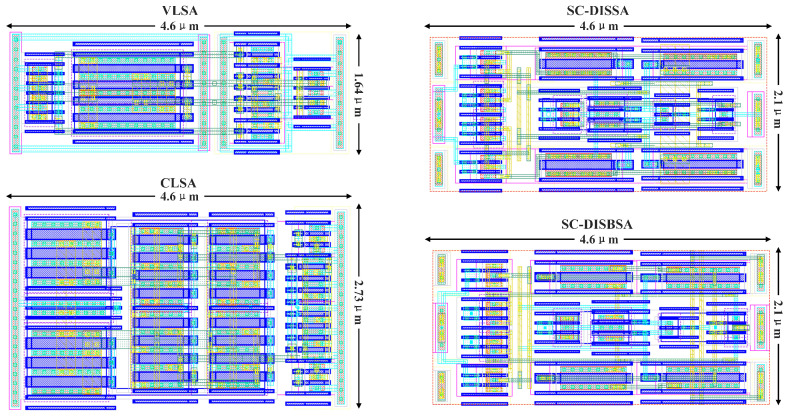
SA layout design optimized for matching the pitch of 8-column 6T bitcells.

The layout depicted in Figure 18 accounts for the matching between SAs and the adopted SRAM architecture. Since 8-to-1 read column multiplexers are employed, each SA is designed to match an 8-bitcell column pitch (4.6 μm) along the gate-width direction. Given that offset is highly sensitive to layout symmetry, the design shown in Figure 18 maintains strict symmetry in both device placement and metal routing to ensure consistent parasitic parameters at critical nodes. Despite incorporating well taps, a strategic floorplan allows SC-DISBSA to achieve a compact area of 9.66 $\mu m^{2}$, identical to that of SC-DISSA. The areas of VLSA and CLSA are 7.54 $\mu m^{2}$ and 12.56 $\mu m^{2}$, respectively. Regarding routing complexity, VLSA utilizes metal layers M1–M3, whereas the other structures require M1–M4. In terms of gate area alone, VLSA is 28% larger than SC-DISSA and SC-DISBSA. Based on the Pelgrom mismatch model, if the proposed SAs were increased to match the VLSA gate area, the $\sigma_{\mathrm{OS}}$ could be further reduced by 17.8%. Additionally, the calibration mechanism requires the control circuit depicted in Figure 7. The shared area (shaded in gray) of the calibration control circuit is 40.1 $\mu m^{2}$, while the non-shared area is 24.4 $\mu m^{2}$.

### 3.4. Comparison with State of the Art

Table 1 compares the proposed SAs with current state-of-the-art designs. A differential-input body bias SA (DIBBSA-PD) [29] based on a 65 nm CMOS process achieves a $\sigma_{\mathrm{OS}}$ of approximately 14.3 mV at VDD = 1.0 V, representing a 26.5% improvement over VLSA. However, its offset significantly exceeds that of SC-DISBSA and SC-DISSA, making it more suitable for low-voltage, low-power SRAM applications. The CLSA in achieves a $\sigma_{\mathrm{OS}}$ of 4.99 mV using cancellation based on delay and offset relation [34], which is nearly double that of SC-DISBSA. Despite a similar area overhead, the single-ended offset-canceling SA (SOSA) [35] exhibits considerably higher $V_{\mathrm{OS}}$ and energy consumption over the proposed designs. The body-biased hybrid SA (BHSA) [36] leverages multi-threshold devices and a positive feedback mechanism to achieve a $\sigma_{\mathrm{OS}}$ of 3.2 mV at 0.9 V, whereas the proposed designs offer further reductions in offset. The offset-tolerant body-biased SA (OTB-SA) [37] employs a slow-rising-edge control technique to mitigate mismatch effects. While effective in reducing area overhead for achieving comparable $\sigma_{\mathrm{OS}}$, OTB-SA significantly constrains SRAM read speed.

In summary, the proposed SC-DISSA and SC-DISBSA schemes utilize an unconventional BL power strategy to achieve low power consumption and reduced leakage. By leveraging a gate-voltage-modulated self-adaptive calibration technique for quantitative mismatch compensation, these designs offer low offset. Consequently, the proposed SAs significantly optimize the read delay and read energy efficiency of SRAM. Furthermore, they hold promise for application as high-precision comparators in emerging computing-in-memory architectures.

## 4. Conclusions

Based on the linear relationship between $V_{\mathrm{OS}}$ and the transmission-gate voltage, this study constructs a three-step self-adaptive calibration model and proposes a low-offset SC-DISBSA along with its topological variant. By utilizing a calibration control circuit for quantitative $V_{\mathrm{OS}}$ adjustment, SC-DISSA improves $V_{\mathrm{OS}}$ by 55.6% compared to the uncalibrated state. Furthermore, SC-DISBSA employs dynamic body-biased SA calibration to further suppress adverse mismatch or leverage favorable mismatch, achieving an additional 9.9% $V_{\mathrm{OS}}$ improvement over SC-DISSA. Simulation results verify that across 0.7 V to 1.1 V, the $\sigma_{\mathrm{OS}}$ of SC-DISBSA and SC-DISSA remains stable below 3.1 mV and 3.3 mV, respectively. This significantly optimizes SRAM read delay and energy consumption. Compared to VLSA, our designs reduce the required $T_{\mathrm{BL}}$ for $\Delta V_{\mathrm{BL}}=6\sigma_{\mathrm{OS}}$ by at least 42%. Additionally, SC-DISBSA and SC-DISSA exhibit clear advantages in $T_{\mathrm{SA}}$, $P_{\mathrm{ST}}$ and $P_{\mathrm{RS}-\mathrm{ave}}$. In conclusion, our designs provide a low-offset and energy-efficient sensing solution for high-speed memory applications.

## Figures and Tables

**Table 1:** Comparison with state-of-the-art works.

	TCAS-I [29]	Microelectron. J. [34]	TCAS-II [35]	TCAS-II [36]	TCAS-I [37]	This Work	
	DIBBSA-PD	CDOR-CLSA	SOSA	BHSA	OTBSA	SC-DISSA	SC-DISBSA
Technology	65 nm	28 nm	28 nm	22 nm FDSOI	28 nm	28 nm	
Supply (V)	1.0	0.9	0.8	0.9	1.0	0.9	
devices	9T	15T	16T+2C	13T	7T	14T	
Gate area ($\mu m^{2}$)		/	/		0.73	0.52	
	15.6% reduction ^*a*^			3.9% reduction ^*a*^	61.4% reduction ^*a*^	18.9% reduction ^*a*^	
Layout area ($\mu m^{2}$)	11.5	/	9.2	7.32	7.72	9.66	
	1.5% reduction ^*a*^			14.8% increase ^*a*^	49.6% increase ^*a*^	31.3% increase ^*a*^	
$\sigma_{\mathrm{OS}}$ (mV)	14.3 ^*c*^	4.99	6	3.2	4.98	2.9	2.59
	26.5% increase ^*a*,*c*^	71.4% reduction ^*b*^		47.5% reduction ^*a*,*c*^		49.3% reduction ^*a*^	54.7% reduction ^*a*^
	28.1% reduction ^*b*,*c*^			59.5% reduction ^*b*,*c*^		65.8% reduction ^*b*^	69.5% reduction ^*b*^

^*a*^ Compared to the VLSA implemented in respective work. ^*b*^ Compared to the CLSA implemented in respective work. ^*c*^ Based on approximate readings from data figures from the original paper.

## Data Availability

Data are contained within this article.
